# Exhaustive Search of the Receptor Ligands by the CyCLOPS (Cytometry Cell-Labeling Operable Phage Screening) Technique

**DOI:** 10.3390/ijms21176258

**Published:** 2020-08-29

**Authors:** Irina A. Ishina, Ioanna N. Filimonova, Maria Y. Zakharova, Leyla A. Ovchinnikova, Azad E. Mamedov, Yakov A. Lomakin, Alexey A. Belogurov

**Affiliations:** 1 Shemyakin-Ovchinnikov Institute of Bioorganic Chemistry RAS, 117997 Moscow, Russia; iraishina94@gmail.com (I.A.I.); ioannishka@gmail.com (I.N.F.); marusya3@mail.ru (M.Y.Z.); leyla_ovchinnikova@yahoo.com (L.A.O.); bioaz12@gmail.com (A.E.M.); 2 Pirogov Russian National Research Medical University, 117997 Moscow, Russia; 3 Lomonosov Moscow State University, 119991 Moscow, Russia

**Keywords:** antigen-specific B cell, filamentous phage, fd bacteriophage, M13 bacteriophage, BCR, immunoglobulin selection, FACS, ligand–receptor interaction, MHC II, HLA-DRB1

## Abstract

Effective and versatile screening of the peptide ligands capable of selectively binding to diverse receptors is in high demand for the state-of-the-art technologies in life sciences, including probing of specificity of the cell surface receptors and drug development. Complex microenvironment and structure of the surface receptors significantly reduce the possibility to determine their specificity, especially when in vitro conditions are utilized. Previously, we designed a publicly available platform for the ultra-high-throughput screening (uHTS) of the specificity of surface-exposed receptors of the living eukaryotic cells, which was done by consolidating the phage display and flow cytometry techniques. Here, we significantly improved this methodology and designed the fADL-1e-based phage vectors that do not require a helper hyperphage for the virion assembly. The enhanced screening procedure was tested on soluble human leukocyte antigen (HLA) class II molecules and transgenic antigen-specific B cells that express recombinant lymphoid B-cell receptor (BCR). Our data suggest that the improved vector system may be successfully used for the comprehensive search of the receptor ligands in either cell-based or surface-immobilized assays.

## 1. Introduction

Despite the remarkable advances made by molecular biology techniques, determining the specificity of the antigen receptors is still a non-trivial and challenging task. Designing a reliable technique that will be able to identify the B and T cell specificity could help elucidate the etiology of many human diseases and discover new therapeutic antibodies and vaccines. Various screening approaches to investigating the antigen-specific cells have been undertaken in the last few years. Novel technological breakthroughs in microfluidics made it possible to select the immunoglobulins with distinct specificities from the Fab (antigen binding fragments) [1] or scFv (single-chain variable fragment) [2,3] libraries with natively paired heavy and light chains. Alternatively, the 10 × Genomics platform was used by Goldstein and colleagues for the high-throughput sequencing of the antigen-specific individual B-cell receptors (BCR), located directly on the living B lymphocytes, that recognize the desired protein [4]. Finally, LIBRA-seq developed by Setliff et al. allows identifying the BCRs for several barcoded antigens simultaneously [5]. The main limitation of the described techniques is the restricted number of the antigens that can be analyzed in a single experiment, as each individual protein should be expressed, purified, and labeled separately. Thus, a library of the bacteriophages that would simultaneously carry up to millions of the DNA-encoded ligands seems to be a beneficial alternative to the barcoded recombinant proteins.

Studying the T cells specificity requires the new human leukocyte antigen (HLA) ligands to be determined. The etiology of many infectious and autoimmune disorders is associated with the specificity of HLA class II, which provides protection from or, on the contrary, increases susceptibility to a disease. At present, the majority of such specific antigens are still unknown [6]. The existing computer-based algorithms used to predict peptide binding to the HLA of different alleles [7] often result in unreliable calculations. Mass spectrometric analysis of the peptides eluted from the HLA molecules essentially allows identifying its sequence [8]. Nonetheless, this methodology works properly for the over-represented peptides, whereas rare ligands are highly likely to be overlooked. Several protocols that describe the identification of the HLA peptide ligands by phage display [9,10] have been reported previously. A direct analysis of the specificity of HLA exposed on the living cell by phage display is still a challenging task. The diminished number of surface HLA molecules, even in the case of the professional antigen-presenting cells (APC) [11], significantly limits the interpretation of the obtained data. Therefore, the ongoing improvement of the phage display techniques that could provide a robust selection of the HLA peptide ligands in one round will help to carry out an exhaustive screening of the custom antigen libraries, associated with specific disorders.

The possibility to express various peptides on the surface of bacteriophage particles as a fusion protein with the p3 protein was demonstrated by George Smith in 1985 [12]. Among the five coat proteins of filamentous M13, f1, or fd bacteriophage, the p3 and p8 proteins are the most frequently used carriers in the phage display technology [13]. The bacteriophage p3 protein is exploited for the N-terminus or, less frequently, for the C-terminus fusion with the peptide, scFv or cDNA libraries [14,15,16,17]. The fusion with the p8 protein, in contrast to the p3 protein, makes it possible to present thousands copies of ligands on a single phage particle. Nonetheless, a huge amount of the p8 protein may oppositely result in a crowded ligand conformation, which leads to a significant loss of the binding activity [18]. Alternative approaches include using the p6 protein for the C-terminus fusion [19] or p7 and p9 bacteriophage proteins for the N-terminus presentation [20].

The expression of ligands fused with the p3 bacteriophage protein is provided by either phage or phagemid vectors. The phagemid vector is relatively smaller in comparison with the phage vector and thus can accommodate a larger DNA fragment. The combinatorial libraries are often assembled on the basis of phagemid vectors, since it has been noticed that they have a higher transformation efficiency [21]. However, in the case of the phagemid, an additional helper phage such as M13K07 [22] is required to produce the functional phage particles carrying the exposed ligand. To increase the percentage of the phage particles exposing the ligand of interest, different variants of the modified helper phages (hyperphage, KM13, R408d3, Ex-phage, Phaberge, Superphage, etc.) were proposed [23,24,25,26,27]. Their operating principle consists in impairing the functioning of the wild-type p3, which predominantly promotes the packaging and secretion of the recombinant p3 protein fused with the ligand. In contrast to phagemids, the phage vector contains all the genes necessary for the replication and assembly of the bacteriophage [28]. This results in a multivalent display of the fusion protein, i.e., 3–5 copies per each single phage particle. Such phages have a significantly higher avidity, compared to the phages generated by the phagemids and the helper phages where ligand is exposed on 1–5% of the phages in the singular [23,29].

In our recent studies, we showed the possibility to select the antigen-specific B cells using a combination of the engineered bacteriophage bioconjugates and flow cytometry technique with an unprecedented selectivity reaching 97% [30,31]. This approach allows us to screen thousands of ligands in a single experiment. The investigated system is more efficient, sensitive, processive, and low-cost in comparison to selecting via synthetic biotinylated peptides. To bypass the labor intensive large-scale production of the hyperphage or other modified helper phage, we aimed to develop the phage system to identify the antigen-specific cells where there is no need to use helper phages. In this study, we compared the efficiency of phage- and phagemid-based systems for the selection of antigen-specific cells. Similar to the previously published pHen2-based phagemid constructs [30,31], we created a publicly available fADL-1e-based phage vector, containing a cassette for the integration of ligand and a detection tag. Here, we show that the developed system is not only highly efficient in selecting the antigen-specific cells using flow cytometry, but is also beneficial for identifying ligands of the peptide-HLA-TCR trimolecular complexes.

## 2. Results

### 2.1. Engineering of the fADL-1e Phage Vector for the Efficient Production of Bacteriophages Carrying Exposed Ligands

**Figure 1:**
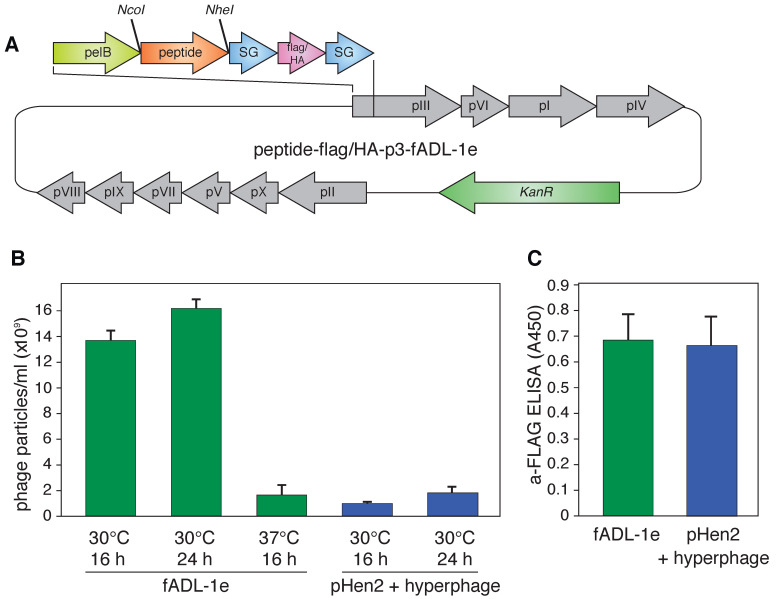
(**A**) Vector map of the fADL-1e-based plasmid for the bacterial expression of the fd filamentous bacteriophage with the p3 protein N-terminally fused with peptide ligand and 3xFLAG or HA-tags flanked by the SG-linkers. The full vector sequences are available online (Addgene ID: 139441 (3xFLAG) and Addgene ID: 139440 (HA)); (**B**) Yield of the bacteriophage expression by the fADL-1e- and pHen2-transformed cells after 16 or 24 h of incubation at 30 or 37 °C; (**C**) Level of the 3xFLAG-p3 in the bacteriophages (10^9^ particles/mL) produced by the fADL-1e- or pHen2-hyperphage-based systems measured by ELISA.

The phage fADL-1e vector derived from the fd-kan, a type 3 phage display vector, was used as a genetic basis for the CyCLOPS protocol that aimed at detecting the antigen-specific cells. The fADL-1e vector contains the full phage genome, thus, it does not require a helper phage to produce virions. We assembled fADL-1e-flag-linker-p3 (Addgene ID: 139441) and fADL-1e-HA-linker-p3 (Addgene ID: 139440) vectors that contain p3-N-terminally fused cluster encoding the 3xFLAG or HA (hemagglutinin) epitopes for detection, serine-glycine linkers for the conformational flexibility, and *Nco*I and *Nhe*I restriction sites for the subsequent cloning of the oligonucleotides coding for the custom peptide ligands (Figure 1A). As a model for the ligand–receptor interaction, we used transgenic Raji-FL cells that express membrane-tethered BCR (B-cell receptor) in a single-chain format, derived from a patient with follicular lymphoma cells, and its peptide ligand P#1 (CILDLPKFC) [30,32] cloned into the phage vector (P#1-HA-p3-fADL-1e or P#1-flag-p3-fADL-1e). Our data suggest that the fADL-1e-based phage vector increased bacteriophage yield by an order of magnitude in comparison with the pHen2-based phagemid vector and hyperphage infection (Figure 1B). Instruction manual for fADL-1 phage vectors (Antibody Design Laboratories) recommends overnight incubation at 37 °C for virion production. In our experiments, we observed that incubation at 30 °C significantly elevate bacteriophage production. The fADL-1e-transformed cells produce more than 10^10^ of the bacteriophage particles already after a 16-h incubation at 30 °C. The analysis of the bacteriophages generated by the fADL-1e- and pHen2-transformed cells revealed that the level of exposed 3xFLAG peptide per phage particle was equal (Figure 1C).

### 2.2. Utilization of the fADL-1e-Based Protocol Significantly Decreases False-Positive Staining of the Antigen-Specific B Cells

**Figure 2:**
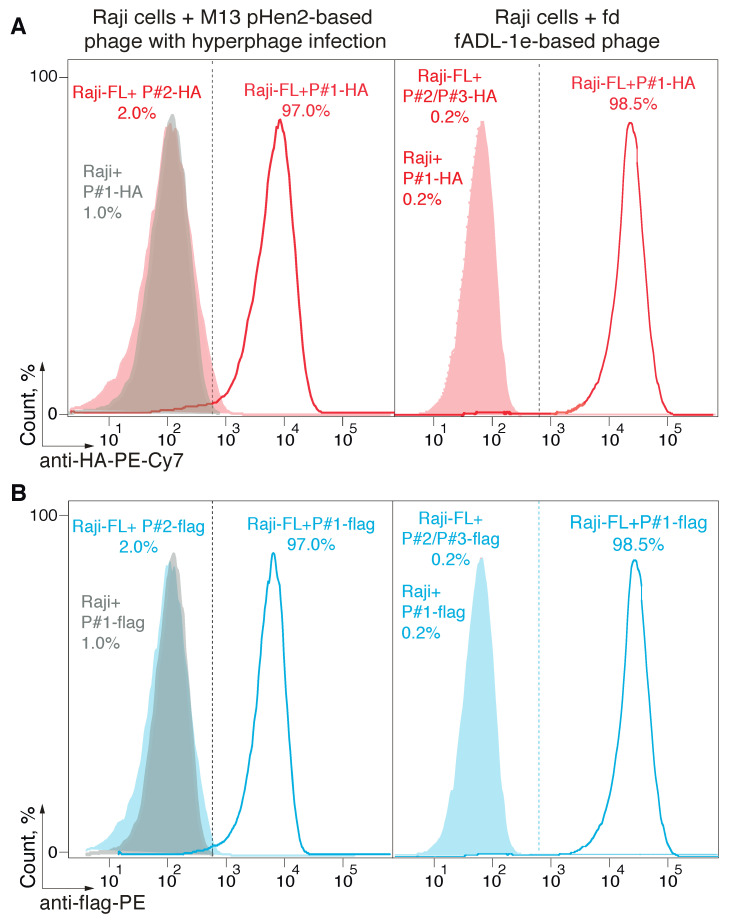
Detection of the receptor-ligand interaction with the fADL-1e-based and pHen2-produced bacteriophages. Ligand–receptor interaction was studied by the flow cytometry using Raji cells expressing a membrane-tethered BCR in a single-chain format (Raji-FL cells), and filamentous bacteriophages carrying its peptide ligand (P#1). Non-transduced Raji and Raji-FL cells were incubated with filamentous phages exposing P#1-peptide fused with HA-tag (**A**) or 3xFLAG (**B**) at a concentration of 1 × 10^12^ or 5 × 10^12^ phage particles per mL for fADL-1e-based (**right**) or pHen2-based (**left**) protocols, respectively. Phages exposing irrelevant P#2 and P#3 peptides were used as a negative control. Fluorescence signals are plotted on the *x*-axis, and the percentage of the recorded events is on the *y*-axis. Histograms for pHen2-based + hyperphage system (**left**) show the cut-off gate at a false-positive signal level of 2%. Histograms for fADL-1e-based (**right**) show the cut-off gate at a false-positive signal level of 0.2%.

Further, we tested if a fADL-1e- or pHen2-based protocol generates functional phage particles that can be used for the CyCLOPS technique. The ligand–receptor interaction was studied following the previously developed methodology [30]. Raji cells that express membrane-tethered BCR in a single-chain format (Raji-FL) were stained with its BCR peptide ligand (P#1) exposed on the surface of filamentous bacteriophages produced via phage fADL-based or phagemid pHen2-based vectors. The phage particles were used in a concentration range of 2 × 10^11^–5 × 10^12^ bacteriophages per mL. The binding of bacteriophages to the eukaryotic cells was detected by incubation with the anti-HA-PE-Cy7 (Figure 2A) or anti-flag-PE (Figure 2B) fluorescent antibodies, which was followed by flow cytometry. Similar to the results obtained previously [30], usage of the hyperphage and pHen2-based vector allowed detecting 97.0% of antigen-specific cells with a 2% false-positive signal level, which corresponded to a control experiment with Raji-FL cells stained with bacteriophages that carried an irrelevant peptide P#2. The application of the fADL-1e-produced bacteriophages with either HA- or 3xFLAG-tag at a range of concentrations from 2 × 10^11^ to 5 × 10^12^ phage particles per mL allowed us to confidently select 98.5 ± 0.4% of Raji-FL cells with a 0.2% false-positive signal level. These data confirm the possibility of the fADL-1e-based vectors to produce bacteriophages, which are highly suitable for the antigen-specific selection of the eukaryotic cells by flow cytometry. It may be suggested that the fADL-1e-based phage vector is more beneficial as compared to pHen2 phagemid not only in terms of its productivity, but also an increased separation efficiency.

### 2.3. CyCLOPS Is Configured to Simultaneously Analyze up to the Ten Thousand Individual Antigens

**Figure 3:**
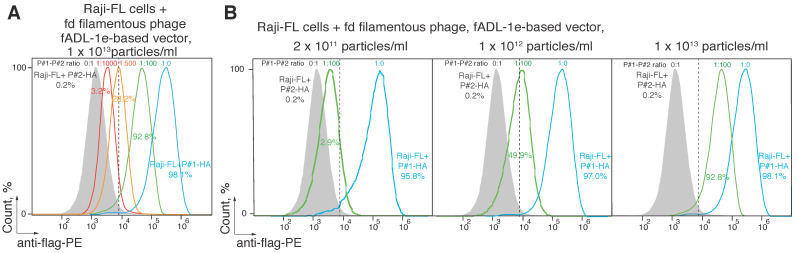
Estimation of the minimal concentration of the ligand-carrying bacteriophage sufficient for the successful CyCLOPS technique. Ligand–receptor interaction was studied by the flow cytometry using Raji cells expressing a membrane-tethered BCR in a single-chain format (Raji-FL cells) and filamentous bacteriophages carrying its peptide ligand (P#1). Raji-FL cells were incubated with filamentous phages exposing P#1-peptide, irrelevant P#2 peptide, or a mixture of phages carrying P#1 and P#2 peptides in ratios of 1:1000, 1:500, and 1:100 at a final concentration of 1 × 10^13^ (**A**) or in a ratio of 1:100 and concentration of 2 × 10^11^–1 × 10^13^ phage particles per mL (**B**). Fluorescence signals are plotted on the *x*-axis, and the percentage of the recorded events is on the *y*-axis. Each histogram shows the cut-off gate at a false-positive signal level of 0.2%.

Next, we tested if it is possible to select specific ligands of the cell surface receptors utilizing the developed CyCLOPS platform. We mixed bacteriophages, exposing specific (P#1) and non-specific (P#2) peptides, in ratios of 1:10,000, 1:1000, 1:500, and 1:100 at the final concentration of 1 × 10^13^ phage particles per mL (Figure 3A). Staining the Raji-FL cells with a hundredfold predominance of the phages that carry non-specific ligand allowed us to select 92.8 ± 1.2% of the antigen-specific cells with a level of the false-positive signal 0.2%. Further reduction of the ratio of P#1- to P#2-carrying phages to the values of 1:500 and 1:1000 resulted in the decrease of the reliably selected antigen-specific cells to 28.2 ± 1.4% and 3.2 ± 1.1%, respectively. Conversely, diluting the bacteriophages, which carry specific peptide P#1, ten thousand times with irrelevant bacteriophages showed no statistically significant cell staining (data not shown). Additionally, we revealed that the minimal phage concentration of 2 × 10^11^ particles/mL of a hundredfold diluted mix is sufficient for selecting 2.9 ± 0.6% of antigen-specific B cells (Figure 3B). A fivefold elevation of the concentration of mixed bacteriophages to 1 × 10^12^ particles/mL allowed us to confidently sort 49.9 ± 1.5% of the antigen-specific cells, wherein elevation of phage concentration to 1 × 10^13^ particles per mL allowed selecting 92.8 ± 1.2% of the targeted cells and still did not increase unspecific binding.

To validate the CyCLOPS technique further, we amplified the sequences coding for the peptides fused with the p3 protein from the positively sorted Raji-FL cells that were incubated in the P#1/P#2-carrying bacteriophage mixture 1:100 at a concentration of 2 × 10^11^ (P#1 concentration 2 × 10^9^) or 1 × 10^12^ (P#1 concentration 1 × 10^10^) phage particles per mL (Figure 3B). Additionally, we analyzed the unsorted Raji-FL cells incubated in the same bacteriophage mixture. The obtained PCR-products were ligated into the DNA vector and after that 15 clones from each sorting experiment were sequenced (Figure S1). As a result, all 15 clones from the positively selected bacteriophages contained P#1 peptide, while all clones from the unsorted mixture contained irrelevant P#2 peptide. Thus, we can conclude that the preferable concentration of the individual bacteriophage in CyCLOPS technique should exceed 2 × 10^9^ phage particles per mL with optimal range starting from the 1 × 10^10^ phage particles per mL.

### 2.4. CyCLOPS May Be Adopted for the Selection of the HLA Class II Ligands

**Figure 4:**
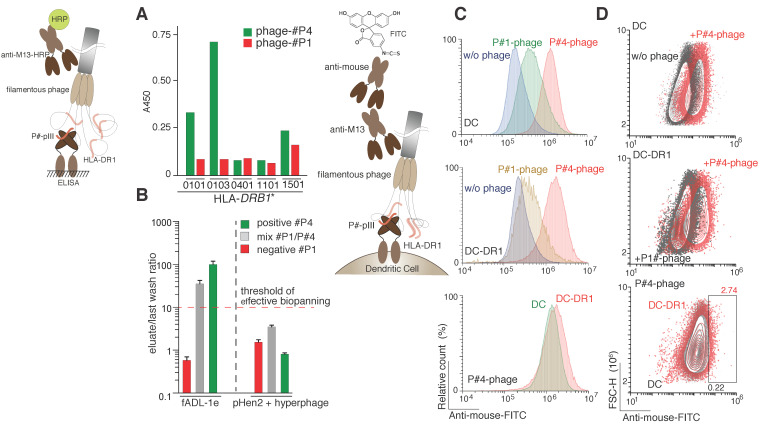
Selection of the HLA class II ligands. (**A**) Binding of peptide ligands exposed on the phage particles by various recombinant HLA-DRB1 molecules (0101, HLA-DRB1*0101; 0103, HLA-DRB1*0103; 0401, HLA-DRB1*0401; 1101, HLA-DRB1*1101; 1501, HLA-DRB1*1501) measured by ELISA; (**B**) The HLA class II peptide epitope selection by fADL-1e-based (left) and pHen2-based (right) phage display. Ligand–receptor interaction was studied by incubating the recombinant HLA-DR1 (in complex with HLA-DM) with its peptide ligand (P#4) itself or in mixture with irrelevant peptide P#1 (P#4/P#1 ratio 1:100) exposed on the surface of the filamentous bacteriophage. Phages exposing only irrelevant P#1 peptide were used as a negative control. Afterwards, the phage-HLA complexes were captured by anti-MHC II antibodies. After extensive washing steps, the *E. coli* TG1 cells were infected with the eluted phages. The CFU of the last wash and eluate were estimated for each selection experiment. Ratio of the CFU of eluate versus last wash (*n* = 3) are plotted on the logarithmic *y*-axis. A ratio greater than one order of magnitude indicates effective selection; (**C**,**D**) Cell-based detection of the HLA II–ligand interaction with the fADL-1e based bacteriophages by flow cytometry. Non-transduced dendritic cells (DC) and dendritic cells transduced with HLA-DRB1*01:01 (DC-DR1) were incubated with filamentous fADL-1e-based bacteriophages exposing P#1 or P#4 peptides at a concentration of 7 × 10^12^ phage particles per mL. Fluorescence signals are plotted on the *x*-axis, and either the percentage of the recorded events (histograms) (**C**) or FSC-H (contour plots) (**D**) is on the *y*-axis.

Identifying the novel MHC class II ligands in vitro is a tricky task due to the solubility problems and conditions that cannot imitate the native environment. Previously, it was reported that the truncated form of the MHC class II has a better solubility in water solutions [33], which makes it possible to maintain the most structural features of the MHC II. Thus, the recombinant form of the human MHC class II, namely HLA-DR1 (HLA-DRB1*01:01), with reduced transmembrane domains of both alpha and beta chains fused with the Jun-Fos leucine zipper, was used as a receptor. To maintain the native-like environment for the HLA class II peptide loading during in vitro conditions, the recombinant HLA-DM was used as a catalytic chaperone of the HLA-DR1 peptide exchange [34]. The potential HLA epitopes were exposed on the surface of the phage particles belonging to the fADL-1e- or pHen2-based phage libraries. The peptide of the hemagglutinin protein of the *influenza A virus* P#4 (PKYVKQNTLKLAT) [35] was chosen as a model specific ligand, while irrelevant peptide P#1 (CILDLPKFC) was used as a negative control. The verification of binding of recombinant HLA class II molecules with its peptide ligands, exposed on the phage surface, was accomplished by ELISA (Figure 4A). We performed three sets of the HLA-DR1 epitope selection for both fADL- and hyperphage pHen2-based protocols: (i) phages carrying only P#4 peptide; (ii) P#1-carrying phages; and (iii) mixture of P#4/P#1-carrying phages in a 1:100 ratio. One single round of the biopanning for all the three variants was accomplished. After the interaction of the phage particles with HLA-DR1, the phage-HLA complexes were captured by the anti-HLA antibodies, pre-immobilized on immunotubes. After extensive washing steps, the bound phages were eluted and subsequently infected the *E. coli* TG1 cells.

Significant difference between value of the colony forming units (CFU) detected in the last washing step and elution samples was observed only for the fADL-based system already after the first round of selection. A noticeable CFU increase up to two orders of magnitude was detected in elution fractions in comparison with the appropriate last washing step fractions in the case of the biopanning with P#4-carrying phage and a mixture of the P#4/P#1-carrying phages (Figure 4B). The biopanning with P#1-carrying phages revealed almost no clones in the eluate, suggesting a low level of the non-specific HLA–phage interaction. In the case of the pHen2-based hyperphage protocol, no significant difference of the CFU presence in the last washing step and eluate fraction was observed. We further analyzed 20 clones after the first round of biopanning from the each of the following samples: (i) initial phage mixture for the fADL-based system; (ii) eluted phages from the mixture of fADL-based phages; (iii) initial phage mixture for the pHen2-based system; and (iv) eluted phages from the mixture of pHen2-based phages. Finally, we observed that all the analyzed clones eluted from the mixture of fADL-based phages (ii) were identified as the positive control peptide P#4, wherein no other positive clones were detected in all other samples (Figure S2). These data suggest that the developed fADL-1e-based phage system is the most efficient for the HLA class II peptide epitope screening. Cell-based assay demonstrated specific binding of the fADL-1e phages exposing P#4 peptide in comparison with those carrying irrelevant P#1 peptide with dendritic cells expressing recombinant HLA-DR1 (Figure 4C,D). Nonetheless, we observed that P#4-peptide fADL-1e phages also significantly bind non-transduced dendritic cells. This may be explained by the ability of the hemagglutinin-derived P#4 peptide to bind almost all DRB1 molecules, which was shown previously [36,37] and in the present study (Figure 4A). The murine cells DC2.4 expose murine MHC II, that may also bind phages with P#4 peptide. Thus, we may suggest that the efficiency of HLA class II ligand screening in the format of the biopanning seems to be relatively higher than in cell-based assay.

## 3. Discussion

Various methods of studying the antigen-specific lymphocytes, e.g., cytometry and emerging mass cytometry, are available at the present time. Nonetheless, this kind of analysis requires laborious labeling of each individual antigen with a fluorophore or rare metal isotope, thus reducing the number of the analyzed antigens. The mass cytometry technique has an incredibly high phenotyping resolution; however, this methodology functions in a *post mortal* mode and does not presuppose the living cells sorting. On the one hand, in the present report we demonstrated that combining the phage display technique and FACS can dramatically improve the screening of the peptide ligands receptors with an unknown or complex structure in their natural environment.

The proposed CyCLOPS platform may also be utilized to select antigen-specific lymphocytes, allowing to identify the neutralizing antibodies for treating both viral and bacterial infections, cancer, inflammation disorders, etc. This platform may be especially useful in the fight against a new rapidly growing epidemic, when the exact immunogenic determinants are yet not known [38]. In these cases, it is possible to use peptide library of viral proteome in order to determine the structure of virus-specific B-cell receptors from the patients who have successfully overcome the infection. CyCLOPS platform may be effectively used for elucidation of the etiology of the autoimmune diseases, in which there is no clear understanding what autoantigens are involved in the disease triggering. Our estimation suggests that routinely CyCLOPS platform may provide analysis of the 10^4^ individual phage-exposed peptides of 50 a.a. long, which covers the majority of the human proteome associated with the autoimmunity. It should be emphasized here that evident disadvantage of all phage-based systems including CyCLOPS is restricted ability to select antigen-specific cells recognizing conformational epitopes assembled from the amino acid residues distanced from each other in the linear sequence. The CyCLOPS platform may be also used for the multidimensional screening purposes, namely: (i) to screen library of surface cell receptors versus library of phage-exposed peptides; or (ii) to use different phage libraries (e.g., carrying different tags for independent detection) in order to select surface receptors recognizing several peptide ligands simultaneously.

The reliable method for detecting novel antigens may potentially lead to developing vaccine candidates and elucidating the disease etiology. Previously, we have reported the high-throughput platform that can be used for this purpose [30]. Despite its efficient selectivity, the protocol that was developed beforehand required the use of the only commercially available helper phage—hyperphage. Due to its high cost, the use of the helper hyperphage in large-scale experiments is troubled. In the current study we advance the previously reported technique [30] and deploy the publicly available phage fADL-1e-based vectors, encoding all genes necessary for assembling the virion particles. In the designed vectors the p3 protein is fused with the cluster containing 3xFLAG or HA-tag for the detection, serine-glycine linkers, and restriction sites for the insertion of the custom peptide library. Importantly, CyCLOPS protocol does not require the antigen biotinylation step that can potentially lead to its aggregation and precipitation. The fADL-1e-based protocol exhibited the higher yield of virion expression compared to the pHen2-based system. We showed that the developed fADL-1e-based bacteriophage system is more effective than the hyperphage pHen2-based one for the purpose of selecting the antigen-specific cells. Moreover, the fADL-1e-based protocol revealed a high specificity of the assembled phages in ligand–receptor interaction tests both ex vivo and in vitro. Here, we also report that CyCLOPS may be successfully used for the HLA class II peptide epitope screening. To conclude, the current study demonstrates the power of CyCLOPS not only in terms of selecting the antigen-specific cells with an unprecedented efficacy, but also when it comes to the uHTS of millions of compounds specific to diverse cell surface receptors in their native environment, as well as in vitro native-like conditions.

## 4. Materials and Methods

### 4.1. Cell Lines

The Raji-FL cell line with membrane-tethered BCR in a single-chain format, that specifically binds the P#1 peptide (CILDLPKFC), was obtained earlier by stable transduction of Raji cells [32]. Raji and Raji-FL cells were cultured in RPMI medium (Gibco, OK, USA) supplemented with 10% (*v*/*v*) fetal bovine serum (Cytiva, MA, USA) and L-glutamine (Gibco, OK, USA) at 37 °C, 5% CO_2_.

The genetic constructs for recombinant HLA-DRA1*01:01 and HLA-DRB1*01:01 (HLA-DR1) were created on the base of lentivirus pLV vector. The constructed vector carried α and β HLA-DR chains fused with T2A peptide for the self-cleavage between the chains during translation. Dendritic cells (DC2.4 cell line) were transduced with pLV-HLA-DR1 genetic construct using standard protocol. The stable cell line was obtained by labeling with anti-HLA-DR-mAb (L243) and goat pAb to mouse IgG (DyLight550) and subsequent enrichment on Sony SH800 FACS cell sorter. The cell-surface expression of HLA-DR1 molecules was verified by FACS analysis (Figure S3).

### 4.2. Plasmids

All original plasmid constructs are accessible through the Addgene plasmid repository (https://www.addgene.org). The fADL-1e-HA-linker-p3 (Addgene ID: 139440) and fADL-1e-flag-linker-p3 (Addgene ID: 139441) vectors are based on phage fADL-1e vector (Antibody Design Laboratories, San Diego, CA, USA) with the addition of linker sequence that was fused with the p3 protein of fd filamentous bacteriophage and encodes an HA-tag (hemagglutinin tag) or 3xFLAG for detection, serine-glycine linkers for conformational delimitation, and *Nco*I and *Nhe*I sites for the cloning of the desired peptide sequences. We used the following peptides: P#1—CILDLPKFC—positive ligand for Raji-FL cells; P#2—HKLIHAASERVLSDARTILEENIQDQDVLLLIKKRAPSPLPKMA—irrelevant protein; P#3—PTCNIRVTVCSFDDGVDLPP—irrelevant protein; and P#4—PKYVKQNTLKLAT—positive control substrate for HLA-DR1 peptide fragment of *influenza A virus* hemagglutinin [35]. PCR was performed on a Thermal cycler T100 (BioRad, Berkeley, CA, USA) using Tersus polymerase (Evrogen, Moscow, Russia).

### 4.3. Expression and Purification of Recombinant Bacteriophages

The recombinant phages were obtained under standard protocol for hyperphage [23] or fADL phage [39] expression system. Briefly, the phage fADL-based vectors were transformed into *E. coli* TG-1 and seeded on a Petri dish (LB-agar, 50 μg/mL kanamycin, 2% glucose). The colonies were inoculated into 5 mL of a 2xYT medium containing 50 μg/mL kanamycin, 2% glucose and incubated at 37 °C overnight. The overnight culture was diluted 1:100 in a 2xYT medium containing 50 μg/mL kanamycin and incubated at 30 °C for 16–24 h. The phagemid pHen2-based vectors were transformed into *E. coli* TG-1, seeded on Petri dishes (LB-agar, 100 μg/mL ampicillin, 2% glucose). The colonies were inoculated into 5 mL of a 2xYT medium containing 100 μg/mL ampicillin, 2% glucose and incubated at 37 °C overnight. The overnight culture was diluted 1:100 in a 2xYT medium containing 100 μg/mL ampicillin, 2% glucose, and grown at 37 °C to a density OD_600_ = 0.5, after which 10 mL of the culture (4 × 10^9^ bacterial cells) were infected with hyperphage M13K07ΔpIII (Progen, Germany) in a ratio of bacterial cells to helper phage 1:20 and then incubated for 30 min at 37 °C without shaking. The cells were precipitated by centrifugation at 3000× *g* for 10 min, the precipitates were resuspended in 200 mL of a 2xYT medium with 100 μg/mL ampicillin and 50 μg/mL kanamycin, grown for 16–24 h with intensive shaking at 30 °C.

Phages were purified from the overnight culture with double PEG precipitation described previously [30]. The counting of the number of colony-forming units (CFU/mL) was performed by transduction, while the determination of all bacteriophages (including non-infective) concentration (particles/mL) was performed by ELISA.

### 4.4. ELISA

MaxiSorp 96-well plates (Nunc, Thermo Fisher Scientific, Waltham, MA, USA) were coated with 0.25 μg/well anti-M13 antibodies (Sigma-Aldrich, St. Louis, MO, USA) in 100 mM carbonate buffer (pH 9.0) at 4 °C overnight, and washed with PBS, containing 0.1% Tween 20 (pH 7.4). This washing was performed after each step. The wells were then blocked with 5% dry milk in carbonate buffer and incubated for 1 h at 37 °C. The plates were incubated with analyzed bacteriophages diluted in PBS containing 0.05% Tween 20 and 0.5% dry milk at 37 °C for 1 h; then, respective monoclonal HRP-conjugated antibodies were added in dilution 1:3000 (anti-HA-HRP, Sigma-Aldrich, USA) or 1:5000 (anti-flag-HRP; anti-M13-HRP, GE Healthcare, Amersham, UK), and the plates were incubated again at 37 °C for 1 h. After washing, tetramethyl benzidine (Amresco, Solon, OH, USA) was added to each well, and the plates were placed in the dark for 10 min. The reaction was stopped by adding 10% phosphoric acid. The A_450_ values were measured on a Varioskan Flash microplate reader (Thermo Fisher Scientific).

### 4.5. Staining of Antigen-Specific Cells with Bacteriophages Followed by Flow Cytometry

Staining of antigen-specific eukaryotic cells by bacteriophages exposing peptide ligands was performed under protocol described previously [30]. Briefly, 0.5 million Raji and Raji-FL cells were resuspended in 100 μL of PBS with 0.5% bovine serum albumin and 2 mM ethylenediaminetetraacetic acid (EDTA), containing bacteriophages with P#1-P#4 peptides exposed on its surface in the final concentrations from 2 × 10^11^ to 4 × 10^12^ particles/mL. Then, the samples were incubated for 1 h at 4 °C with constant shaking and washed three times with PBS containing 2 mM EDTA. Anti-flag-PE or anti-HA-PE/Cy7 fluorescent antibodies (1:500, Biolegend, San Diego, CA, USA) were added to the samples and incubated in the dark for 30 min at 4 °C. Unbound fluorescent antibodies were washed away twice with PBS containing 2 mM EDTA. At each step, cells were centrifuged at 4 °C and 300× *g* for 5 min and all reagents were pre-cooled to 4 °C. Fluorescence intensity was measured with a flow cytometer (Cell Sorter SH800, Sony Biotechnology, San Jose, CA, USA) at excitation/emission wavelengths 565/578 or 496/785 nm. All data were processed using Flow Jo software (Tree Star, Ashland, OR, USA).

### 4.6. Enrichment and Sequencing of Antigen Ligands Exposed on Bacteriophage Surface

Raji-FL or Raji cells were mixed with the bacteriophages exposing P#1, the bacteriophages exposing P#2 or the mixture of bacteriophages exposing P#1 and P#2 in ratio 1:100 and 1:10000. The cells were stained with bacteriophages as described above and sorted using fluorescence-activated cell sorting (FACS). The sorted cells with bounded bacteriophages were kept at −20 °C until further experiments. The nucleotide sequences of the selected peptides fused with p3 were amplified by PCR with pelB forward primer (5′-GATTGTTATTACTCGCGGCCCAGCCGG-3′) specific to pelB leader sequence and reverse flag primer (5′-ATCACCGTCATGGTCTTTGTAGTC-3′) annealing to flag epitope sequence. The resulting PCR amplicons were ligated in the pal2t vector (Evrogen, Moscow, Russia). The ligation mixture was transformed in XL-1 *E. coli* cells, then 20 random colonies from each sorting insert were amplified by PCR with M13 forward (5′-GTAAAACGACGGCCAGT-3′) and M13 reverse (5′-AGCGGATAACAATTTCACACAGGA-3′) primers (Figure S1), and the selected peptides were sequenced.

### 4.7. HLA Preparation

Recombinant MHC II HLA-DR1, obtained from *Drosophila melanogaster* cell line S2, was isolated, as described previously, by a sequential FPLC purification using anti-MHC II affinity column and ion-exchange column Mono-Q [40,41]. The purity grade was more than 80%. Recombinant HLA-DM, containing IgG Fc domain for stability and purification, was produced in eukaryotic cell line HEK293-F, purified in two stages on protein G affinity column (GE Healthcare) and MonoQ column [40,41]. All proteins were stored for no longer than 1 month at 4 °C.

### 4.8. Selection of Peptide Substrates on HLA II by Phage Display

The bacteriophages exposing positive control substrate for HLA-DR1 - peptide fragment of *influenza A virus* hemagglutinin (P#4) [35], the bacteriophages exposing negative peptide (P#1) and the mix of positive/negative control phages P#4/P#1 (1:100 ratio) were used to estimate the efficacy of HLA-DR1 peptide substrate selection by phage display technique and to compare the phage fADL-based and the phagemid pHen2-based systems. The equimolar quantities of recombinant HLA-DR1 and HLA-DM (50 pmol) were mixed with 2.5 × 10^9^ phages in citrate buffer (50 mM Na citrate, 150 mM NaCl, 2 mM EDTA, 0.2% NP40, 1 mM PMSF, pH 6) in 50 µL and incubated at 25 °C during 24 h. Simultaneously immunotubes (Nunc) were coated with 2 mL of anti-MHC II antibodies L243 (12.5 µg/mL) at 4 °C during 24 h and then blocked with 5% dry milk in PBS. Each mixture of phage:HLA-DR1:HLA-DM, diluted by citrate buffer with 0.5% dry milk up to 1.5 mL, was loaded into an immunotube coated with anti-MHC II-antibodies. A separate immunotube was loaded with phage:HLA-DM mixture without MHC II. After a 2-h incubation at room temperature without shaking, the multiple sequential washing steps with 5 portions of 4 mL of PBS with 0.1% Tween 20 and 10 portions of 4 mL of PBS were performed. Each wash step was no shorter than 5 min. Then, the elution of the bounded phages was carried out by incubating them with 1.5 mL of 100 mM Gly-HCl, pH 2.5 at 25 °C for 10 min. The eluates were neutralized with 2 M Tris-HCl, pH 7 (200 µL per 1 mL of Gly-HCl solution). CFU (Colony Forming Units) of the eluates were determined by infecting *E. coli* strain TG1 in the log phase (OD_600_ = 0.6) during 30 min without shaking and followed by plating the cultures on LB-agar with kanamycin (for fADL-based phages) or ampicillin (for pHen2-based hyperphages). CFU values were compared between the last washing steps and the eluate fractions for each selection. The percentage of phages exposing positive control peptide P#4 in the obtained mixtures was determined for each selection by colony PCR with P#4 forward primer specific to 5′ terminus of P#4 sequence (5′-ATAGTTCCATGGCCCCGAAATATGTGAAACAGAA-3′) and P3 reverse primer specific to p3 protein sequence (5′-TGATATTCACAAACGAATGGATCC-3′).

### 4.9. Binding Assay of Dendritic Cells, Expressing Recombinant DR1 Molecules with Bacteriophages, Exposing HLA II-Ligands by FACS

Non-transduced and HLA-DR1 transduced murine dendritic cells (DC2.4 cell line) (2 × 10^6^) were fixed with the fixation buffer according to the manufacturer protocol (R&D Systems, Minneapolis, MN, USA). Fixed cells were washed with PBS and resuspended in 200 μl of PBS with 0.5% bovine serum albumin (BSA), 2 mM EDTA, and 0.025% sodium azide (NaN_3_), containing P#1 or P#4 fADL-1e-based bacteriophages in the final concentration of 7 × 10^12^ particles/mL. The samples were incubated at 37 °C for 16 h with constant shaking. Then, the samples were washed three times with PBS containing 2 mM EDTA. Mouse anti-M13 (1:500, GE Healthcare) and anti-Mouse-FITC (1:200, BioLegend, San Diego, CA, USA) antibodies were added in series and incubated in the dark for 40 min at 4 °C with constant shaking. For the assessment of cell-surface HLA expression level anti-HLA-DR-APC (L-243 clone) (1:250, Beckman Coulter, Brea, CA, USA) antibody was added to the dendritic cells and incubated in the dark for 40 min at 4 °C. Unbound antibodies were washed away twice with PBS containing 2 mM EDTA. At each step, cells were centrifuged at 4 °C and 300× *g* for 5 min and all reagents were pre-cooled to 4 °C. Fluorescence intensity was measured with a flow cytometer (NovoCyte Flow Cytometer, ACEA Biosciences, San Diego, CA, USA) at excitation/emission wavelengths 488/530 or 640/675 nm for FITC or APC channels, respectively. All data were processed using Flow Jo software (Tree Star, USA).

### 4.10. Statistical Analysis

All experiments were carried out in triplicates (biological repeat) and all samples were measured at least in triplicates (technical repeat).

## Supplementary Materials

The following are available online at https://www.mdpi.com/1422-0067/21/17/6258/s1, Figure S1: PCR analysis of randomly selected clones obtained after ligation of amplified P#1/P#2 sequences from positively sorted (A,B) or unsored and unwashed (C,D) Raji-FL cells incubated with P#1/P#2-carrying bacteriophages in ratio 1:100 at a concentration of 2 × 10^11^ (A,C) or 1 × 10^12^ (B,D) phage particles per mL in fADL-1e-based system. Insert of nucleotide P#1 sequence in pal2t vector corresponds to the 390 bp PCR-product; insert of nucleotide P#2 sequence in pal2t vector corresponds to the 490 bp PCR-product. (E) Visualization of the difference between PCR-products from P#1 or P#2 sequence in pal2t vector on the same agarose gel. Detailed experiment description is provided in Section 4.6—*Enrichment and sequencing of antigen ligands exposed on bacteriophage surface*. Figure S2: Agarose electropherogram of PCR samples obtained after analysis of randomly selected clones from biopaning of P#4/P#1 bacteriophage mixture against HLA-DR1 utilizing fADL-1e-based (A) or pHen2-based (B) systems and from initial P#4/P#1 bacteriophage mixture obtained with fADL-1e-based (C) or pHen2-based (D) systems. Figure S3: FACS analysis of cell-surface expression of the HLA-DR1 molecules in dendritic cells transduced with HLA-DRB1*01:01 (DC-DR1). The shadowed plot represents non-transduced dendritic cells (DC), while the red plot represents transduced dendritic cells (DC-DR1). The anti-HLA-DR antibody (clone L243) bind α-chain when α/β heterodimer is properly formed. Fluorescence signals are plotted on the x-axis and FSC-H is on the y-axis.
