# Supplementary material for: Exhaustive Search of the Receptor Ligands by the CyCLOPS (Cytometry Cell-Labeling Operable Phage Screening) Technique
Source: Int J Mol Sci. 2020 Aug 29;21(17):6258. doi: 10.3390/ijms21176258 (PMC7504098; doi:10.3390/ijms21176258)
Supplement: Supplementary file 1 [file ijms-21-06258-s001.pdf]

International Journal of
Molecular Sciences

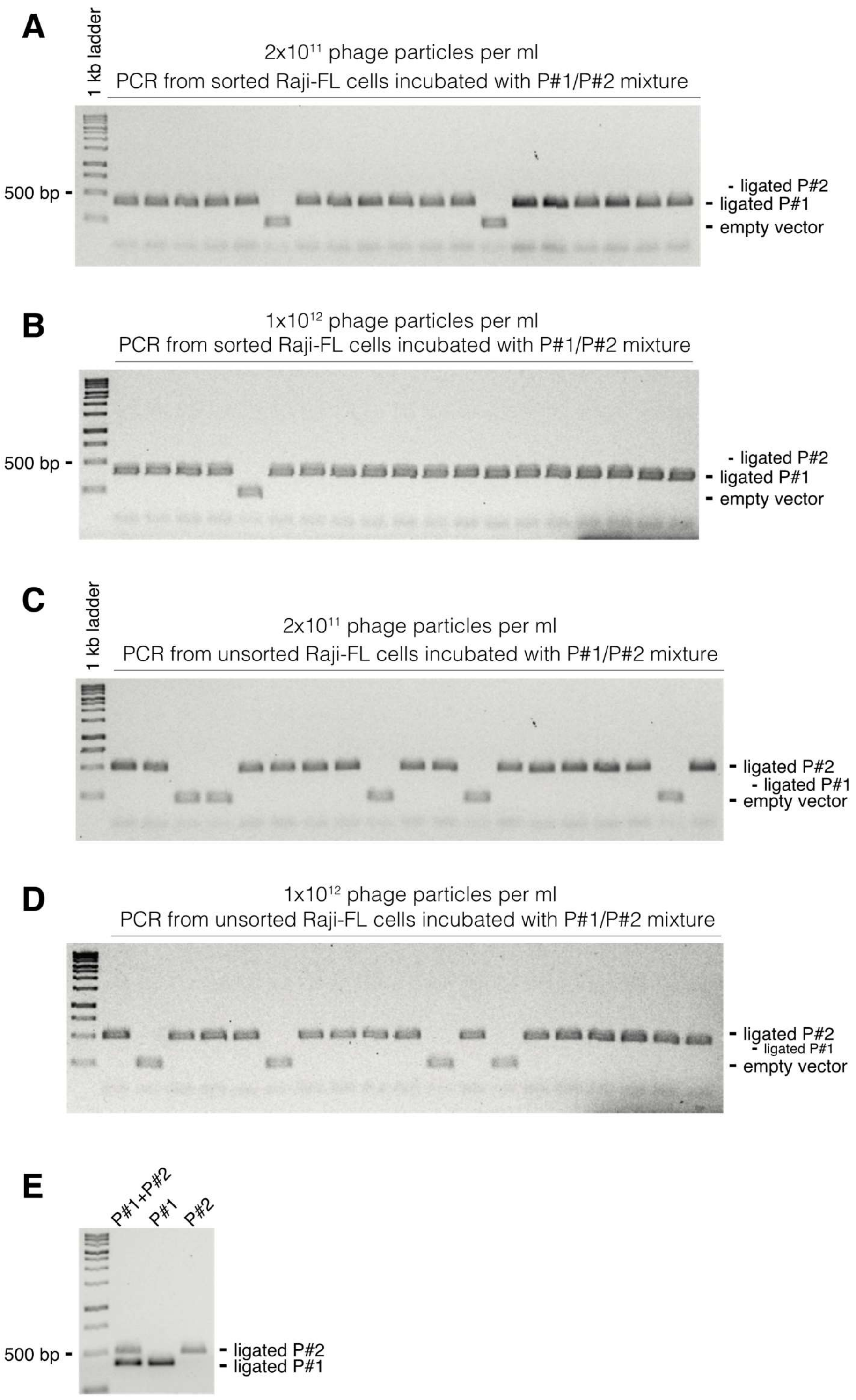

Figure S1. PCR analysis of randomly selected clones obtained after ligation of amplified P#1/P#2 sequences from positively sorted (**A**,**B**) or unsored and unwashed (**C**,**D**) Raji-FL cells incubated with P#1/P#2-carrying bacteriophages in ratio 1:100 at a concentration of 2×10^{11} (**A**,**C**) or 1×10^{12} (**B**,**D**) phage particles per ml in fADL-1e-based system. Insert of nucleotide P#1 sequence in pal2t vector corresponds to the 390 bp PCR-product; insert of nucleotide P#2 sequence in pal2t vector corresponds to the 490 bp PCR-product. (**E**) Visualization of the difference between PCR-products from P#1 or P#2 sequence in pal2t vector on the same agarose gel. Detailed experiment description is provided in Materials and Methods-Enrichment and sequencing of antigen ligands exposed on bacteriophage surface.

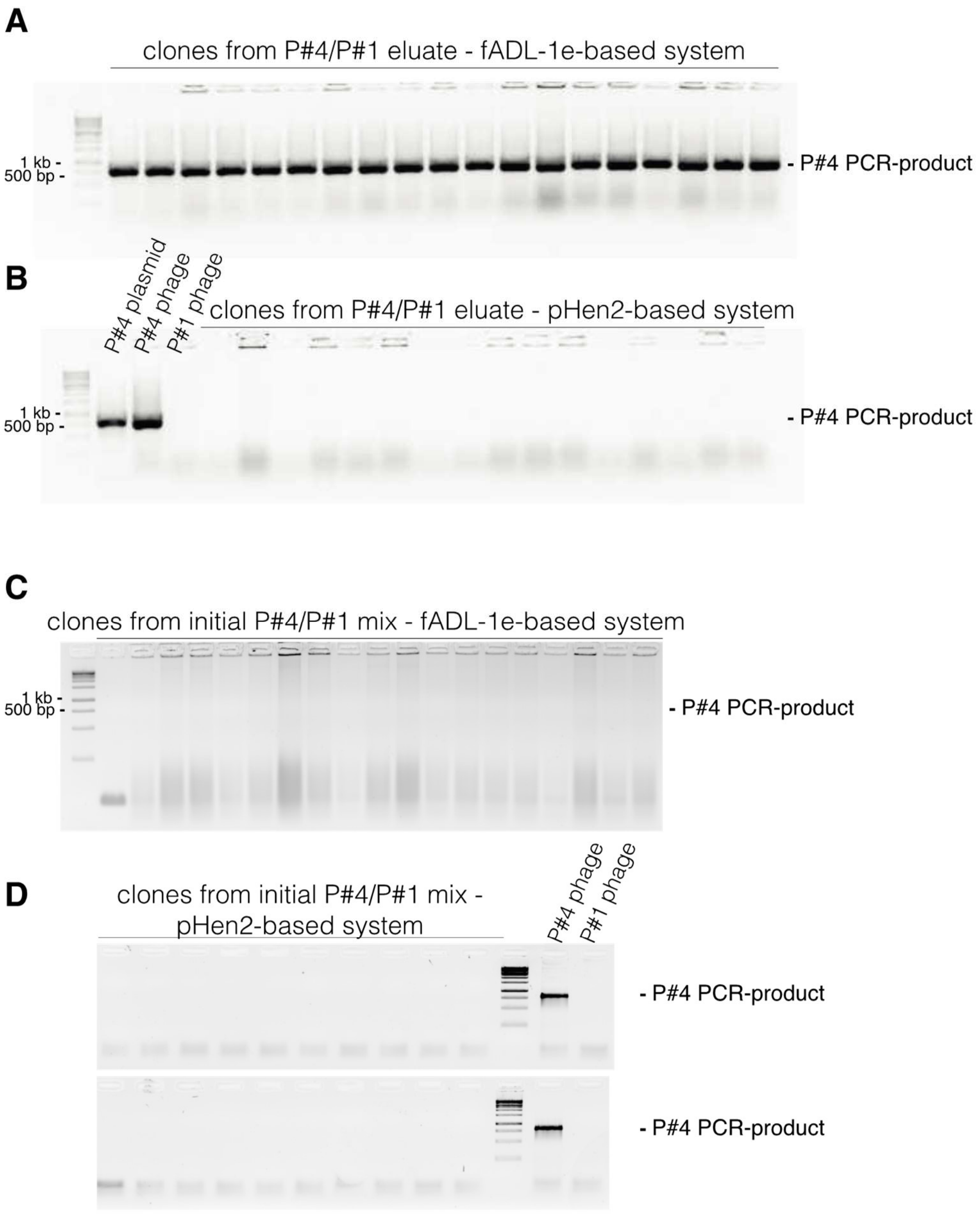

Figure S2. Agarose electropherogram of PCR samples obtained after analysis of randomly selected clones from biopaning of P#4/P#1 bacteriophage mixture against HLA-DR1 utilizing fADL-1e-based (**A**) or pHen2-based (**B**) systems and from initial P#4/P#1 bacteriophage mixture obtained with fADL-1e-based (**C**) or pHen2-based (**D**) systems.

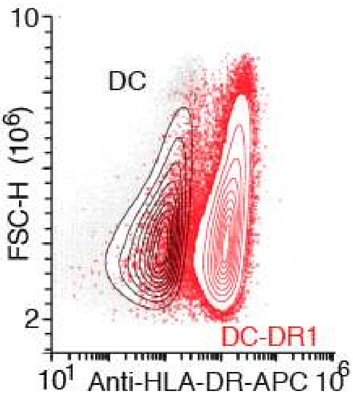

Figure S3. FACS analysis of cell-surface expression of the HLA-DR1 molecules in dendritic cells transduced with HLA DRB1*01:01 (DC-DR1). The shadowed plot represents non-transduced dendritic cells (DC), the red plot represents transduced dendritic cells (DC-DR1). The anti-HLA-DR antibody (clone L243) bind α-chain when α/β heterodimer is properly formed. Fluorescence signals are plotted on the x-axis and FSC-H is on the y-axis.
